# Tunable C_2_N Membrane for High Efficient Water Desalination

**DOI:** 10.1038/srep29218

**Published:** 2016-07-07

**Authors:** Yanmei Yang, Weifeng Li, Hongcai Zhou, Xiaoming Zhang, Mingwen Zhao

**Affiliations:** 1 School for Radiological and Interdisciplinary Sciences (RAD-X) and Collaborative Innovation Center of Radiation Medicine of Jiangsu Higher Education Institutions, Soochow University, Suzhou, China, 215123; 2 School of Physics and State Key Laboratory of Crystal Materials, Shandong University, Jinan, China, 250100.

## Abstract

Water scarcity represents one of the most serious global problems of our time and challenges the advancements in desalination techniques. Although water-filtering architectures based on graphene have greatly advanced the approach to high performance desalination membranes, the controlled-generation of nanopores with particular diameter is tricky and has stunted its wide applications. Here, through molecular dynamic simulations and first-principles calculations, we propose that the recently reported graphene-like carbon nitride (g-C_2_N) monolayer can serve as high efficient filters for water desalination. Taking the advantages of the intrisic nanoporous structure and excellent mechanical properties of g-C_2_N, high water transparency and strong salt filtering capability have been demonstrated in our simulations. More importantly, the “open” and “closed” states of the g-C_2_N filter can be precisely regulated by tensile strain. It is found that the water permeability of g-C_2_N is significantly higher than that reported for graphene filters by almost one order of magnitude. In the light of the abundant family of graphene-like carbon nitride monolayered materials, our results thus offer a promising approach to the design of high efficient filteration architectures.

Because of the surging growth of population, rapid urban development and industrialization progress, the augmentation in available freshwater resources is dwindling in many water-stressed countries of the world^1^. Seawater represents a plentiful resource to compensate the stress of potable water supply through desalination. As one representative desalination methods, reverse osmosis (RO) technique has been commonly adopted^2^. In this strategy, a semi-permeable membrane is placed at the interface between seawater and pure water. Pressure applied at the seawater side forces water flow towards the pure water side, while ions are blocked. The pure water production rate of the RO method is typically low, which is ~0.1 Lcm^−2^day^−1^MPa^−1^ for commercially used RO membranes^3^,^4^. The sustainability of water resource through desalination, therefore, highly depends on the development of new desalination membranes.

A promising approach is graphene filter which is first proposed from computer simulations^3^,^4^,^5^,^6^ and eventually realized in recent experiments^7^,^8^. Thanks to the ultrathin thickness (only one atomic layer) of graphene, the energy consumption in seawater desalination is greatly reduced because water flux scales inversely with the filter thickness. This approach may also works for other two-dimensional (2D) materials, such as such as hexagonal boron nitride^9^, silicene^10^,^11^, phosphorene^12^,^13^,^14^,^15^ and molybdenum disulfide (MoS_2_)^16^. Recently, the MoS_2_-based filter was reported to have high performance given that nanopores are introduced to the MoS_2_ monolayer through bombardment or chemical treatment^17^,^18^. However, to be used as filtering membranes, these materials face the same limitation: they do not have inherent nanopores for water flow and thus post-treatment is essential. The shape and size of the nanopores generated during nanoengineering process are decisive factors for water transparency and ion selectivity. However, the successful experimental realization of proper nanopores still remains challenging at present, which becomes to be a major obstacle to the design of advanced filteration architectures based on these 2D materials.

It is noteworthy that there are various types of graphinic carbon nitrides with inherent nanopores in different shapes, which are potential filteration material candidates^19^,^20^,^21^,^22^,^23^,^24^. In particular, the nanopores in the recently-synthesized graphene-like carbon nitride^25^ (referred to as g-C_2_N, as shown in Fig. 1) has diameter of ~4.1 Å (determined by the N atoms and minus the diameter of a N, ~1.5 Å); the size is very close to that of the water molecule (~4 Å). The C-C and C-N covalent bond-based framework result in excellent mechanical properties of the g-C_2_N which is almost comparable to graphene. The stability of g-C_2_N monolayer at high temperature has also been demonstrated experimentally^25^. Thus, the intrisic porous structure implies high prospect of using porous g-C_2_N monolayer as desalination filter without post-treatment to generate nanopores.

In this work, we report the seawater desalination performance of the g-C_2_N membrane through molecular dynamics (MD) simulations and first-principles calculations. It is found that tensile strain can effectively modulate the permeation of water through the g-C_2_N filter continuously from “closed” to “open” states. The water permeability is about two orders of magnitude higher than the commercial RO membranes, while ion transmission is totally blocked. The high water transparency and vigorous salt filtering capability are attributed to the steric hindrance and electrostatic interactions on the translocations of water molecules and ions through nanoscaled confinements. Our results thus highlight the significance of a new 2D material family for high performance seawater desalination filters.

## Methods

### MD simulations

**Figure 1:**
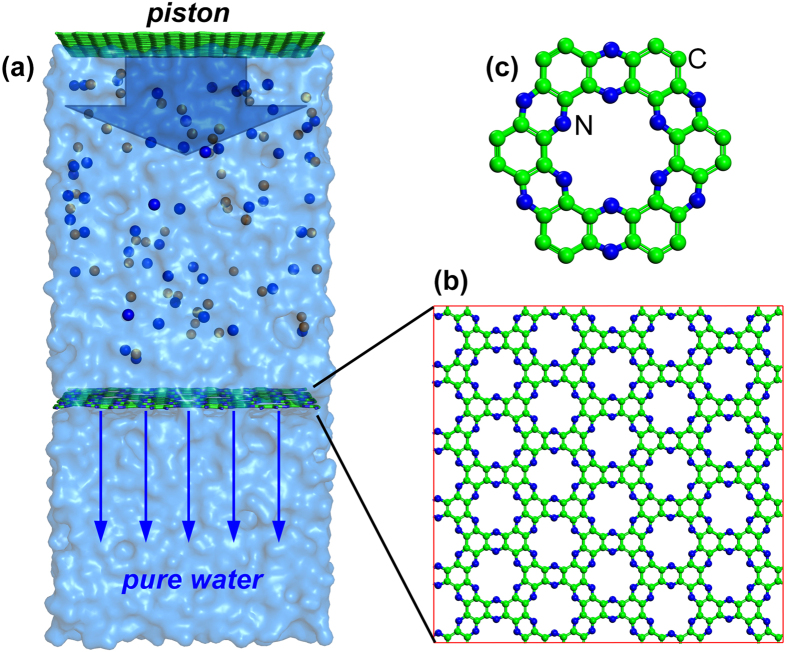
(**a**) Illustration of the simulation model; (**b**) Top view of the nanoporous g-C_2_N layer; (**c**) Local structure of one nanopore in g-C_2_N.

As illustrated in Fig. 1a, the MD simulation model is composed of a seawater region and a pure water region, separated by a g-C_2_N filter. A graphene sheet mimicing the piston is placed on the top of the seawater and force is loaded on it (at 20, 40, 60, 80 and 100 MPa pressure equivalents) in the *z* direction to push water flow towards the pure water region. The g-C_2_N filter (Fig. 1b) containing 30 nanopores has a dimension of roughly 4.16 × 4.33 nm^2^ at the strain-free state. The structure of nanopore is illustrated in Fig. 1c, for which the framework is composed of *sp*^*2*^ hybridized C and pore edges are terminated by N atoms. Strain on the g-C_2_N filter is modulated by increasing and fixing the size of the cross sectional area.

All the simulations were performed with the GROMACS package^26^. The AMBER03 force field^27^ was used in the simulations. The seawater region contains 50 Na^+^ and Cl^−^ ions, and 7000 TIP3P water molecules^28^, corresponding to a salt concentration of 27 g L^−1^, slightly lower than the salinity of seawater (~35 g L^−1^). A lower salinity was chosen for the consideration that water flowing towards the pure water region will effectively increase the salinity in seawater side during the simulation. For the g-C_2_N filter, the atom types of CA and NB were assigned to C and N atoms. The RESP point charges were calculated using the Gaussian 09 code^29^ at the HF/6–31G* level, yielding a value of 0.24 |e| and −0.48 |e| for C and N atoms, respectively. SHAKE constraints^30^ were applied to all bonds involving hydrogen atoms. The long-range electrostatic interactions were treated with the Particle Mesh Ewald method^31^,^32^, and atypical distance cutoff of 12 Å was adopted for the van der Waals (vdW) interactions. The non-bonded interaction pair list was updated every 10 fs. The cross section in the *x-y* plane of the simulation box was fixed to a certain value in order to mimic the strained filter. The box was coupled to a constant at 1.0 atm only along the *z* direction. Canonical sampling was performed through velocity rescaling method at constant temperature 300 K^33^. A movement integration step of 1 fs was used in the simulations. Each system was first equilibrated for 10 ns, followed by 300 ns productive simulation for the data collection.

### First-principles calculations

First-principles calculations were further performed to check the structure stability using the Vienna ab initio simulation package (VASP)^34^,^35^,^36^,^37^. The electron-electron interactions are treated using a generalized gradient approximation (GGA) in the form of Perdew-Burke-Ernzerhof (PBE) for the exchange-correlation functional^38^. The energy cutoff of the plane waves was set to 520 eV with an energy precision of 10^−8^  eV. Vacuum space larger than 15 Å was used to avoid the interaction between adjacent images. The Monkhorst-Pack meshes of 9 × 9 × 1 were used in sampling the Brillouin zone for the g-C_2_N lattice. Tensile strain was applied by fixing the lattice constants to different values. Atomic coordinates were optimized using the conjugate gradient (CG) scheme until the maximum force on each atom was less than 0.01 eVÅ^−1^. Phonon spectra were calculated using a supercell approach within the PHONON code^39^.

## Results and Discussion

### Structural stability of g-C_2_N membrane

**Figure 2:**
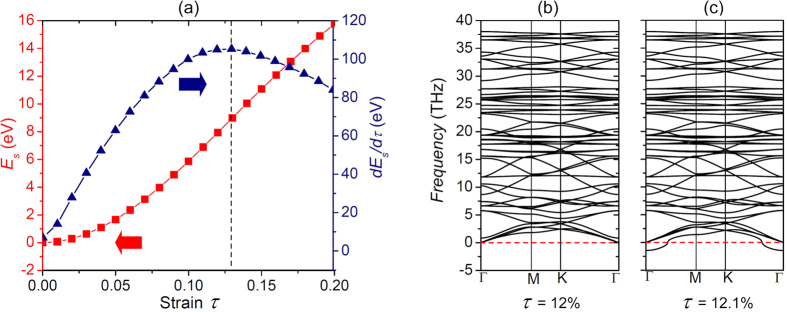
(**a**) Variation of energy in response to tensile strain. (**b**,**c**) Phonon spectra of g-C_2_N under the biaxial tensile strains of 12% and 12.1%. The negative frequencies correspond to the imaginary frequency modes which are dynamically unstable.

The structural stability of g-C_2_N membrane under tensile strain is an important issue. The strain energy (*E*_*s*_) of g-C_2_N under tensile strain (*τ*) was firstly calculated using first-principles calculations. As shown in Fig. 2a, with the increase of tensile strain, *E*_*s*_ increases monotonously as *τ* < 20%. However, it is found that the derivate of strain energy (*dE*_*s*_/*dτ*) reaches the maximum at tensile strain of around 13%, suggesting that the g-C_2_N lattice becomes softer beyond this point. This is futrher confirmed by the features of phonon spectra. When the tensile strain is smaller than 12%, the phonon spectra are free from imaginary frequency modes (Fig. 2b), which indicates the stability of the filter at this critical point. When the tensile strain exceeds 12%, imaginary frequency branch appears as demonstrated in Fig. 2c, which reveals the fact that the g-C_2_N lattice becomes unstable, as external disturbance may destroy the g-C_2_N framework due to the imaginary frequency mode. Both the energy evolution and phonon spectra confirm that the g-C_2_N has excellent mechanical stability which can bear tensile strain of up to 12%. The high structural stability of the g-C_2_N sheets fulfills the requirement of filters working at high pressure.

### Water permeability performance

**Figure 3:**
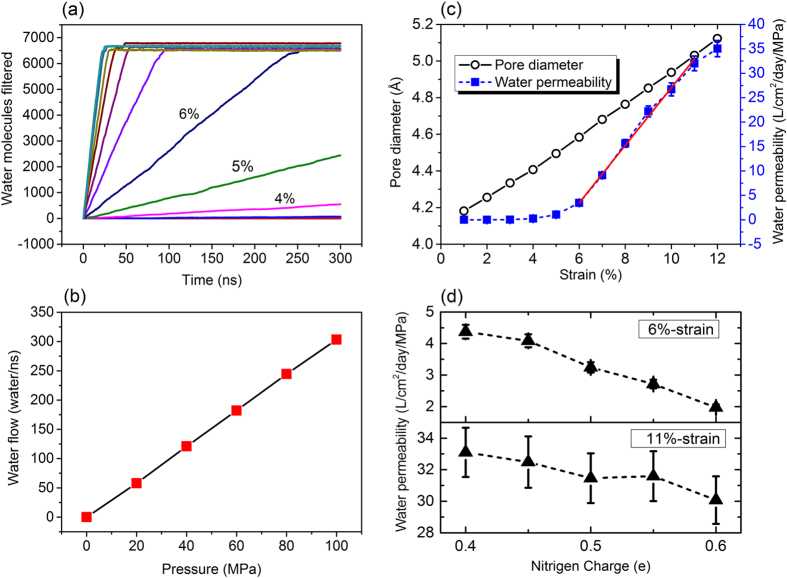
(**a**) Number of water filtered by g-C_2_N membrane as a function of simulation time under piston pressure of 100 MPa, (**b**) Water flow at various pressure through the 12%-stretched g-C_2_N, (**c**) Pore diameter and water permeability with respect to tensile strain and (**d**) Water permeability with respect to the charges of nitrogen of the g-C_2_N filter under tensile strain of 6% and 11%.

We first tested the water transparency of the g-C_2_N filter under the pressure of 100 MPa. The cumulative numbers of water molecules transferred through the g-C_2_N filter are summarized in Fig. 3a. For g-C_2_N filter at the equilibrium state (without tensile strain) and under weakly strained (τ ≤ 1%) conditions, no event of water passage is observed during the entire simulations, indicating that the filter is at “closed” state. The transition point from “closed” to “open” appears at a strain level near 2%, for which 16 water molecules were found to pass through the filter during the 300 ns simulation. As the strain is further increased, the g-C_2_N filter becomes more transparent. For τ ≥ 6%, each curve in Fig. 3a begins with a linear region and eventually reaches a saturation point (around 6500 water molecules) where the entire reservoir of water molecules on the seawater region is completely depleted. This indicates that tensile strain can effectively modulate the permeation of water through the g-C_2_N filter.

Notably, for all the simulations at different tensile strain, the water flow (the slope of curves in Fig. 3a) is constant in time, confirming that well-converged statistics is obtained for all the simulations. The water flow with respect to external pressure for the 12%-strained filter is summarized in Fig. 3b. It is seen that the water flow is proportional to the strength of applied pressure. This allows us to evaluate the water permeability of g-C_2_N filter by extrapolating the dynamic quantities derived here down to the operating condition that is more typical of RO plants (usually several MPa). We expressed the water permeability in liters of output per square centimeter of the filter per day and per unit of applied pressure. As shown in Fig. 3c, it varies from zero (for the unstrained g-C_2_N) to 35.1 Lcm^−2^day^−1^MPa^−1^ (for the extremely strained case). The high performance benefits from the densely packed nanopores in g-C_2_N filter (with density of 1.6 × 10^14^ cm^−2^). For comparison, the highest performance that has been achieved for graphene filter is around 6 Lcm^−2^day^−1^MPa^−1^ which is only 1/6 of that of g-C_2_N filter reported here^7^. It is also noteworthy that the performance of commercial RO is only in the order of 0.1 Lcm^−2^day^−1^MPa^−1 ^5,40. This solidly highlights the importance of the unique porous structure of g-C_2_N as a filter material. More interestingly, there exists a strain window (6–11%) where the water permeability scales almost linearly with strain strength. This property allows a precise control of the g-C_2_N filter which is quite crucial for the design of tunable devices for filtration and other applications.

As illustrated in Fig. 3c, for the 6%-strained filter, the diamater of the permeable pores reaches 4.6 Å, compared to a value of 4.1 Å for strain-free g-C_2_N. For even larger strain, the pore diameter increases almost linearly with respect to the strain strength. The tunable water permeation through the g-C_2_N filter is mainly attributed to the expanded nanopores upon stretching which effectively decrease the steric confinement effect for water passage.

For graphene based filter, it has been evidenced that the edge morphology of the nanopores, especially the hydrophobicity, would significantly regulate the water flow^5^. In view that stretching of chemical bonds may cause slight electron re-distribution between adjacent elements, we examined the effect from atomic charges of the g-C_2_N filter on the water flux. For N, we have considered values from 0.4 to 0.6 |e| in our simulations (the atomic charge of C was changed accordingly). The water permeabilities under tensile strain of 6% and 11% are summarized in Fig. 3d. It is clear that the nanopores with less-charged edge atoms have larger water permeability. On the contrary, accumulation of electrons at the nanopores will decrease the water permeability. In general, the change of water permeability in response to the N charge is only 1.0 Lcm^−2^day^−1^MPa^−1^ per 0.1 |e|, which is much lower than the strain effect. Therefore, it is safe to propose that the regulation of the water flux by strain is mainly determined by the changes of steric hindrance upon modulations of the nanopore size.

### Salt rejection performance

The desalination efficiency is determined by the trade-off between water permeability and salt ion selectivity. Beside high water permeability, a desalination filter should effectively hinder the passage of salt ions. For graphene, the nanopores generated during pre-treatment always have a broad range of diameter, resulting in lower salt rejection performance. For the g-C_2_N, it is exciting that no events of ion passage through the filter have been observed throughout all the simulations with serious of strain and pressure. It is worth noticing that during the simulation, the effective salinity in seawater region keeps increasing because the piston pressure depletes the water molecules in seawater side. This indicates that the salt rejection performance of g-C_2_N filter is robust, which is quite crucial for desalination performance improvement. This should be attributed to the considerable small size of the nanopores, which can efficiently block the ion transmission, corresponding to the maximal water desalination efficiency.

### Free energy profiling analysis

**Figure 4:**
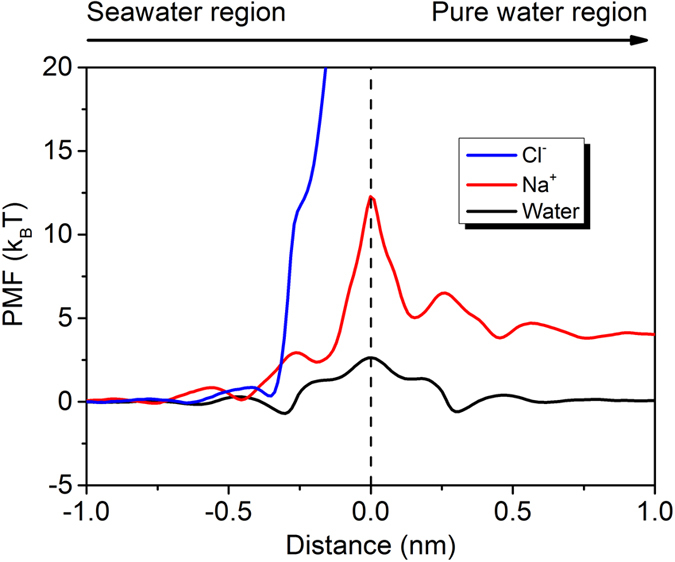
Potential of mean force (PMF) of water, Na^+^ and Cl^−^ passing through the 12%-stretched g-C_2_N filter from seawater region to pure water region. The filter is placed at 0 nm.

The physical origins of the high performance of g-C_2_N filter can be explained by the potential of mean force (PMF) analysis through umbrella sampling. The PMF curves for Na^+^, Cl^−^, and water to across the g-C_2_N filter were obtained by sampling the force experienced by the salt ions or water molecules when passing through the nanopores. Without losing the validity, we have only calculated the PMF curves for the 12%-strained filter. As shown in Fig. 4, the PMF for water molecules is most shallow, with no energy barrier (peak) exceeding 3.32 k_B_T. Typically, an energy barrier of around 5 k_B_T is considered to be low enough for water permeation to happen^41^,^42^,^43^. Hence water molecules can pass through the nanopores easily, leading to high water transparency. Na^+^ is blocked because of the high energy barrier of 12.27 k_B_T near the nanopore center. The probability (*K*_*r*_) for certain solution component to overcome an energy barrier (*E*_*b*_), *K*_*r*_ ∝ exp((−*E*_*b*_)/*k*_*B*_*T*), indicates that the water molecules have approximately 8 × 10^3^ times higher chance to pass through the nanopores compared to Na^+^ ions at room temperature. For Cl^−^ to approach the nanopores from the seawater side, an energy barrier of larger than 35 k_B_T is predicted which is almost inaccessible for transmission to happen.

In experiments, there are several approaches available for applying tensile strain to the 2D monolayer. For instance, g-C_2_N membrane can be deposited on flexible substrate like polymethyl methacrylate. Strain can be directly applied on the substrate to induce deformation of the substrate and accordingly the stretching of the g-C_2_N. Such approach has been adopted for the tuning of electronic structure of MoS_2_ monolayer by strain^44^. Other than direct mechanical stretching, novel substrates which have large thermal expansion coefficient would also serve as the necessary support and introduce strain on the g-C_2_N filter^45^.

## Conclusions

In summary, through molecular dynamics simulations we demonstrate that the recently reported g-C_2_N monolayer can serve as an efficient filter material for seawater desalination. Under tensile strain, the inherent nanopores of g-C_2_N sheets can conduct water in a high transparency manner, while salt ions are completely rejected. The high water transparency and vigorous salt filtering capability are attributed to the steric hindrance and electrostatic interactions on the translocations of water molecules and ions through nanoscaled confinements. The highest water permeability is about two orders of magnitude higher than the commercially-used RO membranes and six times higher than that reported for graphene-based filters. More importantly, the “open” and “closed” states for water flow can be accurately regulated by applying tensile strain. The advantage of the g-C_2_N filter over graphene filter is the inherent porous framework with permeable pores. The easy regulation of the filter with tensile strain and the precise pressure responsive behavior make the g-C_2_N on the horizon to advance the development of desalination filter. Our results also support the design and proliferation of tunable devices for filtration and other applications working at nanoscale.

## Additional Information

**How to cite this article**: Yang, Y. *et al.* Tunable C_2_N Membrane for High Efficient Water Desalination. *Sci. Rep.* **6**, 29218; doi: 10.1038/srep29218 (2016).
